# Stereotactic Radiosurgery for Patients with Brain Metastases from Hepatopancreaticobiliary Cancers

**DOI:** 10.3390/cancers16091665

**Published:** 2024-04-25

**Authors:** Zhishuo Wei, Priyanka Srinivasan, Ritam Patel, Greg Bednarz, John C. Flickinger, Constantinos G. Hadjipanayis, Ajay Niranjan, L. Dade Lunsford

**Affiliations:** 1 School of Medicine, University of Pittsburgh Medical Center, 200 Lothrop Street, Pennsylvania, PA 15213, USA; 2 Center for Image-Guided Neurosurgery, University of Pittsburgh Medical Center, 200 Lothrop Street, Pennsylvania, PA 15213, USAhadjipanayiscg2@upmc.edu (C.G.H.);; 3 Department of Neurological Surgery, University of Pittsburgh Medical Center, 200 Lothrop Street, Pennsylvania, PA 15213, USA; 4 Department of Radiation Oncology, University of Pittsburgh Medical Center, 200 Lothrop Street, Pennsylvania, PA 15213, USA

**Keywords:** brain metastases, stereotactic radiosurgery, HPB cancers

## Abstract

**Simple Summary:**

Brain metastases from Hepatopancreaticobiliary (HPB) cancers are extremely rare and indicate a very poor prognosis. Patients with HPB cancers often have several months of life expectancy from the diagnosis of the brain metastases. Due to the rarity, there is no established protocol on how to treat brain metastases from HPB malignancies. Stereotactic radiosurgery provides the advantage of out-patient noninvasive management of brain metastases. We aim to retrospectively review the post-SRS outcomes of HPB patients with brain metastases in search for the optimal radiation delivery dose for HPB brain metastases. Our study showed that SRS could achieve a 94.7% local tumor control rate per tumor and newly developed brain metastases could be treated with repeat SRS. The favorable outcome of the present study indicates that SRS is effective in controlling HPB brain metastases and is instrumental in preserving patients’ quality of life.

**Abstract:**

Background: The role of stereotactic radiosurgery (SRS) for patients with brain metastases from hepatopancreaticobiliary (HPB) cancers has yet to be established. The authors present a single-institution experience of patients with HPB cancers who underwent SRS when their cancer spread to the brain. Methods: We surveyed our Gamma Knife SRS data base of 18,000 patients for the years 1987–2022. In total, 19 metastatic HPB cancer patients (13 male) with 76 brain metastases were identified. The median age at SRS was 61 years (range: 48–83). The primary cancer sites were hepatocellular carcinoma (HCC, 11 patients), cholangiocarcinoma (CCC, 2 patients), and pancreatic carcinoma (PCC, 6 patients). The median Karnofsky Performance Score (KPS) was 80 (range: 50–90). Two patients underwent pre-SRS whole-brain fractionated radiation therapy (WBRT) and eight patients underwent pre-SRS surgical resection. All SRS was delivered in single session. The median margin dose was 18 Gy (range: 15–20). The median cumulative tumor volume was 8.1 cc (range: 1.0–44.2). Results: The median patient overall survival (OS) after SRS was 7 months (range 1–79 months). Four patients had documented local tumor progression after SRS at a median time of 8.5 months (range: 2–15) between SRS and progression. Out of 76 treated tumors, 72 tumors exhibited local control. The local tumor control rate per patient was 78.9%. The local tumor control per tumor was 94.7%. Four patients developed new brain metastases at a median of 6.5 months (range: 2–17) after SRS. No patient experienced adverse radiation effects (AREs). At the last follow-up, 18 patients had died, all from systemic disease progression. Conclusions: Metastatic spread to the brain from HPB cancers occurs late in the course of the primary disease. In this study, all deceased patients ultimately died from primary disease progression. SRS is a non-invasive strategy that maximally preserves quality of life, and our results reported favorable outcomes compared to the existing literature. SRS should be considered as one of the primary management strategies for patients with brain metastatic spread from HPB cancer.

## 1. Introduction

Brain metastases from hepatocellular carcinoma (HCC), cholangiocarcinoma (CCC), and pancreatic carcinoma (PCC) are very rare, with incidence rates of 0.2–2.2%, 0.5–1.4%, and 0.3–0.6%, respectively [1,2,3]. The onset of brain metastases from these primary malignancies often represents a late manifestation of the primary disease. The prognosis of patients with brain metastases from these malignancies is poor, with survivals after diagnosis of brain spread ranging between 1 and 8.4 months, 2.5 and 3.7 months, and 3.9 and 7.4 months for HCC, CCC, and PCC, respectively [1,2,4]. In recent years, the development of advanced systemic treatment therapies, and consequently longer potential survivals, has led to the increased recognition of brain metastases from these malignancies [1,2]. There is currently no established guideline for the management of patients with brain metastases of hepatopancreaticobiliary (HPB) origin [5]. Management options include surgical resection, stereotactic radiosurgery (SRS) [6], and whole-brain radiation therapy (WBRT) [1].

SRS is an effective non-invasive primary or salvage strategy for a spectrum of more common metastatic cancers that spread to the brain. Outcomes routinely report 1-year local tumor control rates of >85% [7,8]. In this study, we aim to retrospectively review the post-SRS outcome of brain metastases with HPB origin in the search for the optimal radiation delivery dose for HPB brain metastases.

## 2. Materials and Methods

### 2.1. Patient Characteristics

We performed a retrospective analysis of our prospectively maintained data base of 18,000 patients who underwent Gamma Knife SRS at the University of Pittsburgh Medical Center (UPMC) from 1987 to 2022. Patients were included if they had a pathological diagnosis of HPB primary cancer and if they had metastatic disease to the brain confirmed by radiographic imaging studies including computed tomography scanning (CT; 2.5 mm axial slices) and/or T1 magnetic resonance imaging (MRI; 1.5 mm axial slices) with a contrast agent.

Patient and radiographic imaging characteristics are described in Table 1. All data collection was in compliance with our Institutional Review Board-approved outcome assessment goals. In total, 19 patients (13 males) with brain metastases from HPB primary cancers (11 HCC; 6 PCC; 2 CCC) were identified. The median age of the primary disease diagnosis was 61 years (range: 45–80). The median time from primary diagnosis of the primary carcinoma to brain metastasis was 16 months (range: 0–130 months). The median age at SRS was 61 years (range: 48–83). Active systemic disease was reported in 11 patients. The median KPS at SRS was 80 (range: 50–90). Among 15 patients with available histopathology data, 13 patients had adenocarcinoma, 1 patient had squamous cell carcinoma, and 1 patient had combined adenocarcinoma and squamous cell carcinoma. Pre-SRS management included whole-brain radiation therapy (WBRT) in 2 patients and cytoreductive surgery via craniotomy in 8 patients. Concurrent systemic disease management included cytotoxic chemotherapy (4 patients) and immunotherapy (3 patients). At the time of SRS, 17 patients presented with neurological symptoms or signs.

### 2.2. Radiosurgery Technique

Our previous publications have outlined the technical aspects of Gamma Knife SRS procedures [7,9]. The patients were given intravenous conscious sedation and local anesthetic injections before a stereotactic frame was attached to the patient’s head. A double-dose gadolinium-based contrast agent (gadopentetate dimeglumine 469 mg/mL, 0.2 mL/kg) was administrated prior to performing high-resolution spoiled gradient recalled acquisition in steady-state (SPGRs) brain MRI sequences (1.5 mm axial slices) for tumor localization. Axial fast-spin echo T2-weighted 3 mm whole-brain imaging also was performed. Tumor volumes were contoured during the dose-planning process. Various models of the Leksell Gamma Knife units were used in this study. Patients were discharged home the same day.

Factors used to determine the prescription maximal and tumor margin dose included the primary tumor histopathology, the relationship to nearby critical neurological structures (such as the optic apparatus), tumor volume, and prior exposure to brain radiation. Smaller tumors further away from the critical regions received higher margin doses when feasible.

Seventy-six total HPB metastases underwent SRS, and the median number of brain tumors per patient was 3 tumors (range: 1–16 tumors). The median margin dose and maximum dose were 18 Gy (range: 15–20 Gy) and 32 Gy (range: 18.8–40.1 Gy), respectively. The median isodose was 50% (range 45–80%). The median cumulative tumor volume was 8.1 cc (range: 1.0–44.2 cc) and the median cumulative 12 Gy volume was 13.1 cc (range: 2.2–46.8 cc; Table 2).

### 2.3. Patient Follow-Up

During the first year after SRS, patients underwent MRI at 3-month intervals. If no new brain metastases were detected, the time interval for imaging follow-up thereafter was increased to 6 months. High-definition MRI with and without contrast was performed at each clinical follow-up to determine the tumor response after SRS. The Response Assessment in Neuro-Oncology Brain Metastases (RANO-BM) criteria were used to describe the specific tumor response [10]. Local tumor progression was determined as >20% enlargement of the treated tumor. Complete response was defined as the complete disappearance of all enhancing signals. Partial response was defined as a >50% decrease in tumor size. The tumors that did not fit above categories were defined as stable disease. Distant tumor progression was determined as additional tumor development in regions previously untreated with SRS. Repeat SRS was suggested for patients developing progression for treated or previously untreated tumors. Adverse radiation effects (AREs) included reactive edema, temporary treatment-related reactive enlargement, and delayed tumor cyst development.

### 2.4. Study Endpoints

Overall survival (OS) and local tumor control (LTC) were the primary outcomes of this study. Overall survival was calculated using the time interval between the SRS date and date of last clinical follow-up or date of death. LTC was calculated using the time interval between the SRS date and date of confirmed sustained tumor progression or last radiological follow-up. DTC was calculated using the time interval between the SRS date and date of recognition of a new untreated tumor or last radiological follow-up.

### 2.5. Statistical Analysis

Patient, SRS, and outcome characteristics were recorded and analyzed using Excel version 2021 (Microsoft, Redmond, WA, USA). OS, LTC, and DTC curves and rates were plotted and calculated with Kaplan–Meier statistical analyses conducted on Prism Version 9 (GraphPad, San Diego, CA, USA). Information on tables is represented as mean +/− standard deviation (median [range]) or number (%). All values were rounded to two decimal places.

## 3. Results

### 3.1. Overall Survival

The patient outcomes after SRS are presented in Table 3. The median survival after SRS was 7 months (range: 1–79 months). At the last follow-up, 18 patients had died. All patients’ deaths were related to systemic disease progression. The 6-, 12-, and 24-month survival rates from SRS were 66.7%, 40%, and 20%, respectively. Patients diagnosed with PCC, HCC, and CCC had a median overall survival of 7, 9, and 2 months after SRS, respectively.

### 3.2. Tumor Control and Adverse Radiation Effects

**Figure 1:**
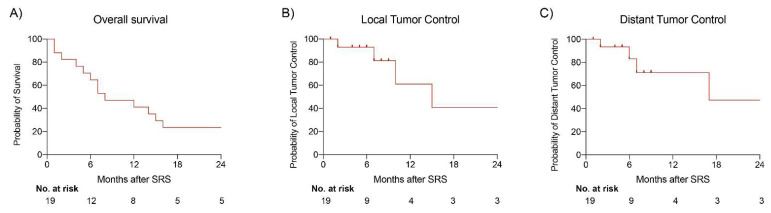
Kaplan–Meier survival curve for HPB brain metastases patients treated with SRS. (**A**) The overall survival was 7 months. (**B**) The overall local tumor control per patient was 78.9%. (**C**) The overall distant tumor control per patient was 78.9%.

In total, four patients (21.1%) displayed local tumor progression despite initial SRS and undergoing repeat SRS. The median time between initial SRS and confirmed treated tumor progression was 8.5 months (range: 2–15 months). Out of the 76 treated tumors, only 4 tumors progressed. The local tumor control per tumor was 94.7%. Four patients (21.1%) developed new brain metastases. The median time between initial SRS and distant tumor progression was 6.5 months (range: 2–17 months). All four patients underwent SRS again. No patients exhibited adverse radiation effects (AREs) after SRS. The Kaplan–Meier survival curves of overall survival, local tumor control, and distant tumor control for HPB patients are presented in Figure 1.

## 4. Discussion

Compared to lung, renal, and breast cancers, HPB brain metastases are rare [1,2]. The improved systemic management of these cancers has resulted in improved survival and recognition of the potential for late brain metastases. In a series of HCC patients, Nam et al. reported a median interval between primary cancer diagnosis and brain metastasis diagnosis of 20.1 months for 86 patients (range: 0–144.1 months) [11]. Similarly, Frega et al. reported five patients who had a median time of 15.7 months (range 7.3–52.8 months) between primary diagnosis and the development of brain metastasis [12]. Jordan et al. reported 24 pancreatic ductal adenocarcinoma (PDAC) patients with a median interval of 17 months between diagnoses in [13]. In the present series, two patients had the rare synchronous diagnosis of both primary and brain metastasis. A single patient only had the primary cancer confirmed after SRS of their brain metastasis. The median interval between primary cancer and brain metastasis diagnoses was 16 months overall (range: 0–130 months). This interval varied between 22, 31, and 9.5 months for HCC, CCC, and PCC, respectively.

### 4.1. Fractionated Whole-Brain Radiation Therapy (WBTR) and Surgery

Before the introduction of SRS, brain metastases from HCC, CCC, and PCC were treated with either primary WBRT or surgical resection, often followed by WBRT. Han et al. [14] reported a median OS of 10.4 weeks for 33 HCC patients after brain metastasis diagnosis. The authors noted that patients who were treated with surgery or surgery after WBRT survived longer (25.3 weeks) as compared to patients who were only treated with steroids. Similarly, Choi et al. [15] retrospectively reviewed 62 HCC patients with brain metastases and reported an OS of 6.8 weeks after the brain metastases diagnosis. The authors noted that five patients who underwent surgical resection followed by WBRT had a median survival of 33.6 weeks (95% CI: 2.0–65.2). In contrast, they noted that 32 patients treated with only surgical resection or only radiation therapy had median survivals of 10.0 weeks. Twenty-five patients who received corticosteroids only survived a median of 2.0 weeks [15]. These results highlight the importance of a multimodality management approach to optimize outcomes in these difficult cancers.

In a series of 19 patients with CCC brain metastases, Dodoo et al. [16] reported a median survival of 6.5 months after surgical resection, radiation therapy, or both as opposed to a median survival of 0.8 months for 4 patients who received only supportive care. D’Andrea et al. [17] found that patients who received fractionated radiation therapy or SRS had improved median survivals (10.2 months, 95% CI: 3.8–16.9) compared to patients treated conservatively (median: 0.8 months, 95% CI: 0.1–1.5). Frega et al. [12] reported six CCC patients whose median survival was 3.7 months (range: 0.9–17.8).

Case series regarding patients with PCC are limited. Matsumoto et al. reported 20 PCC patients who had mean survivals over two years after surgical resection compared to a mean survival of 2.5 months without treatment [18]. Park et al. reported two PCC patients who had no benefit from WBRT.

### 4.2. The Role of Stereotactic Radiosurgery

SRS has emerged as a minimally invasive management that can safely and effectively treat patients with brain metastases in a single-session setting [19,20]. Rare studies have reported the outcomes of HPB brain metastases managed primarily via SRS. The current literature discussed below is summarized in Table 4.

Nam et al. [11] reported an improved median OS of 16.0 weeks in 32 HCC patients who had surgical resection or SRS, compared to a median OS of 4.2 weeks for 54 patients treated with WBRT or conservative care only (*p* < 0.001). However, the OS outcomes for patients treated with solely SRS were not reported by the authors [11]. D’Andrea et al. found a median survival of 6.6 months (range: 3.1–16.9) for four CCC patients treated with SRS [17]. Jordan et al. reported that two patients treated with SRS for PDAC survived for 1.8 and 6.0 months after brain metastases diagnoses [13]. Our study reports an overall median survival time after SRS of 8 months (range 1–79 months). The median survival time after the diagnosis of brain metastasis was 9, 2, and 7 months for HCC, CCC, and PCC primary cancers, respectively. For both HCC and PCC, our median OS values are considerably higher than those in the literature. Our lower-than-reported CCC median OS may be attributed to the small sample size of two patients.

Response to SRS has been related to several factors including tumor histology and tumor volume, history of prior WBRT, and SRS margin dose [21,22]. The current SRS LTC rates for brain metastases from various primary cancers in the literature consistently exceed 85% with a median margin dose range of 18–24 Gy [7,8,23]. However, using a median margin dose of 18 Gy (range: 10–20), Paudel et al. [5] recorded 12-month LTC of 57.21% for 53 patients with brain metastases from only gastrointestinal primary cancers, with increased control rates for higher radiation delivery doses. The median time between SRS and local tumor progression was 4.2 months (range: 1–33.3). In a retrospective study of 23 HCC patients, Han et al. reported that margin doses higher than 18 Gy predict superior outcomes in both univariate and multivariate analyses (*p* = 0.010) [24]. With a median margin dose of 18 Gy (range: 15–20 Gy), our study reported an overall LTC rate per patient of 78.9% and LTC rate per tumor of 94% and a median time between initial SRS and local tumor progression of 8.5 months (range: 2–15). Our overall DTC rate was 78.9% after SRS. For smaller tumor volumes, margin doses of 20 Gy may improve tumor response without increasing the associated risk. However, it would be interesting to analyze the effect of the number of brain metastases, cumulative brain metastasis volume, and the general (KPS) state of the patients, and the effect of systemic therapy in the context of HPB brain metastases after SRS. Due to the rarity of HPB brain metastases, future multi-center studies are warranted for comprehensive analysis.

Compared to patients who undergo WBRT or surgery, it is important to acknowledge that SRS is generally indicated for patients with high KPS scores and better prognostic factors, such as a limited number of brain metastases. Although past studies have demonstrated the safety and efficacy of SRS in patients with an extensive number of brain metastases [7,19,25], those with leptomeningeal disease often receive WBRT. Similarly, patients with large-volume brain metastases causing acute neurological symptoms have a worse prognosis and are not ideal candidates for SRS. These factors partly explain the favorable outcomes observed in the present study. This study emphasizes the importance of carefully selecting ideal candidates with brain metastases of HPB origin for SRS. For the right patients, SRS can be highly beneficial in managing HPB brain metastases.

### 4.3. SRS and Systemic HPB Cancer Therapies

In recent years, the landscape of systemic treatment for hepatobiliary cancers, particularly hepatocellular carcinoma and biliary tract cancers, has evolved significantly. One promising avenue is the potential cooperation between radiotherapy and various systemic treatments. One aspect of interest is the concept of spatial cooperation, where the delivery of SRS or radiotherapy is combined with targeted therapies or immunotherapies, which holds promise in overcoming treatment resistance and maximizing tumor control [26]. Radiation itself has been recognized for its immunogenic effects [27,28], prompting investigations into its role in enhancing systemic immune responses against HPB tumors. These synergistic approaches highlight a dynamic area of research with the potential to further advance the management of HPB cancers and warrant continued exploration in future studies.

### 4.4. Neurological Response

Patients with brain metastases from HPB cancer often present with altered mental statuses, seizures, and headaches. D’Andrea et al. reported that out of the nine CCC patients with brain metastases, three patients presented with seizure, three patients presented with altered mental status, and two patients presented with headaches [17]. Lemke et al. [29] summarized 11 case reports for patients with brain metastases with PCC. Nine of the eleven reported cases had documented the presentation of headaches. In the present study, the median KPS at the presentation of SRS was 80. Only four patients reported worsened symptoms from tumor progression. We acknowledge that this is in part due to the short survival expectance of the patients after relapse. Considering the primary goal should be comfort management, SRS is proved to be a valuable option in managing the intracranial diseases of these patients while maximizing the patients’ remaining quality of life.

### 4.5. Adverse Radiation Effects

ARE rates reported in the literature for brain metastases range from 5 to 20% [30]. Han et al. found that 12.5% of HCC patients with brain metastases treated with SRS experienced CNS radiation-related neurotoxicity [24]. In our study, no patients developed AREs, but this may in part be related to the relatively short survivals of these aggressive cancers. At present, dose prescription represents an attempt to balance tumor control with the risk of AREs [24].

### 4.6. Limitations

This study is primarily limited by its retrospective nature. Further, this study represents the experience of a single institution over a period of 35 years. Patients presenting with HPB brain metastases represent a heterogeneous population and have varied systemic disease treatments. Future multicenter studies are warranted to strengthen our conclusion and elucidate the long-term role of SRS in treating these rare brain metastases of HPB origin.

## 5. Conclusions

SRS represents the best first-line management of HPB cancers that metastasize to the brain. While local brain tumor control was achieved in all patients after one or repeat SRS, survival remained short. In the future, better systemic treatment options coupled with SRS may further improve both total survival and quality of life in these highly aggressive cancers.

## Figures and Tables

**Table 1:** Patient demographics and primary tumor characteristics.

Characteristics	Value
Total No. of pts.	19
Sex	
Male	13 (68%)
Female	6 (32%)
Age at initial primary cancer diagnosis (years)	65 ± 11 (61 [45–80])
Age at initial brain metastasis diagnosis (years)	66 ± 11 (61 [45–83])
Time from primary cancer diagnosis to brain metastasis (months)	16 [0–130]
Age at SRS	67 ± 11 (61 [48–83])
KPS score at SRS	80 [50–90]
Primary histopathology	
Only adenocarcinoma	13 (68%)
Only SCC	1 (5%)
Both adenocarcinoma and SCC	1 (5%)
Data unavailable	4 (21%)
No. of pts. with concurrent systemic therapy	8 (42%)
Cytotoxic chemotherapy (No. of pts.)	4 (50%)
Immunotherapy (No. of pts.)	3 (38%)
Targeted therapy (No. of pts.)	1 (13%)
Prior radiotherapy (RT) characteristics	
Type (No. of pts.)	
None	17 (89%)
WBRT	2 (11%)
No. of pts. with prior surgical resection	8 (42%)
No. of pts. with active primary disease	11 (58%)

No. = number. pts. = patients. KPS = Karnofsky Performance Score. SCC = squamous cell carcinoma. WBRT = whole-brain radiation therapy. Values are represented as either number (%), median [range], or mean ± standard deviation (median [range]).

**Table 2:** SRS planning characteristics.

Characteristics	Value
Metastasis characteristics	
Total no. of HPB metastasis	76
No. of HPB metastasis per pt.	4 ± 4 (3 [1–16])
SRS characteristics	
Margin dose (Gy)	17 ± 2 (18 [15–20])
Max dose (Gy)	31 ± 7 (32 [18.8–40.1])
Isodose (%)	58 ± 13 (50 [45–80])
Cumulative tumor volume of HPB brain metastases (cm^3^) per pt.	12 ± 11 (8.1 [1.0–44.2])
Cumulative V12 (cm^3^) per pt.	18 ± 14 (13.1 [2.2–46.8])

No. = number. pt. = patient. Values are represented as either number (%), median [range], or mean ± standard deviation (median [range]).

**Table 3:** Patient outcomes after SRS.

Characteristics	Value
Median survival (months)	8 (1–79)
Local tumor progression characteristics	
Progression (No. of pts.) at last follow-up	4 (21.1%)
Time between initial SRS and local tumor progression (months)	8.5 (2–15)
Management of local tumor progression (No. of pts.)	
SRS	4 (100%)
Distant tumor progression characteristics	
Progression (# of pts.) at last follow-up	4 (21.1%)
Time between initial SRS and distant tumor progression (months)	6.5 (2–17)
Management of distant tumor progression (No. of pts.)	
SRS	4 (100%)
No. of pts. with ARE	0 (0%)
Median overall survival (months) from SRS	8 (1–33)
Median overall survival (months) from brain metastases diagnosis	8 (1–79)
Cause of death	
Systemic disease	18 (100.0%)

No. = number. pts. = patients. ARE = adverse radiation effects. Values are represented as either number (%), median [range], or mean ± standard deviation (median [range]).

**Table 4:** Prior HPB reports of institutional case series or multi-center studies.

Author/Year	Primary Disease	Management Strategies	# Patients	Time from Primary dx to Brain Mets dx	OS from Brain Mets dx. Median
Nam et al. 2019 [11]	HCC	Resection: 25 WBRT: 37 SRS: 9	86	20 mo. (0–144.1)	1.6 mo.
Han et al. 2013 [14]	HCC	Surgery: 10 WBRT: 12 SRS: 13	33	18.3 mo. (0.5–75)	2.6 mo. (1.3–3.9)
Choi et al. 2009 [15]	HCC	Surgery: 11 WBRT: 16 SRS: 10 Steroids: 25	62	18.2 mo.	1.7 mo.
Jordan et al. 2018 [13]	PCC	Surgery: 4 WBRT: 13 SRS: 3	25	17 mo. (0–79)	1.5 mo.
Frega et al. 2018 [12]	CCC	Surgery: 4 WBRT: 5	6	13.6 mo. (7.3–52.8)	3.7 mo. (0.9–17.8)
Dodoo et al. 2023 [16]	CCC	Surgery: 1 WBRT: 6 SRS: 2	21	14.4 mo.	4.2 mo. (0.2–33.8)
D’Andrea et al. 2020 [17]	CCC	Surgery: 4 WBRT: 5 SRS: 4	9	16.7 mo. (0.7–66.7)	3.8 mo. (0.1–16.9)
Present study	HCC, PCC, CCC	Surgery: 8 WBRT: 2 SRS: 19	Total: 19 HCC: 9 PCC: 7 CCC: 2	16 mo. (0–130)	8 mo. (1–79)

HCC, hepatocellular carcinoma; CCC, cholangiocarcinoma; PCC, pancreatic carcinoma; brain mets, brain metastases; dx, diagnosis; SRS, stereotactic radiosurgery; WBRT, whole-brain radiotherapy; mo., month.

## Data Availability

The data presented in this study are available upon reasonable request to the authors.
